# Enhancing Coordination Skills and Upper-Limb Symmetry Through a Mobile-Application-Based Training Program in 12–14-Year-Old Basketball Players

**DOI:** 10.3390/jfmk11020207

**Published:** 2026-05-25

**Authors:** Steff Norbert, Ioan Sabin Sopa, Dionisie-Vladimir Turcu, Iulian Stoian, Ioan Teodor Hășmășan, Hășmășan Denisa Elena, Sonia Gabriela Neagu, Radu Antonia

**Affiliations:** 1 “Petru Maior” Faculty of Sciences and Letters, “George Emil Palade” University of Medicine Pharmacy Science and Technology, 540142 Targu Mures, Romania; 2 Department of Environmental Sciences, Physics, Physical Education and Sports, “Lucian Blaga” University Sibiu, 550012 Sibiu, Romania; sabin.sopa@ulbsibiu.ro (I.S.S.); dionisie.turcu@ulbsibiu.ro (D.-V.T.); iulian.stoian@ulbsibiu.ro (I.S.); ioan.hasmasan@ulbsibiu.ro (I.T.H.); denisaelena.hasmasan@ulbsibiu.ro (H.D.E.); soniagabriela.neagu@ulbsibiu.ro (S.G.N.); antonia.radu@ulbsibiu.ro (R.A.)

**Keywords:** coordination, basketball, smartphone, bilateral upper-limb performance

## Abstract

**Background**: Smartphones are an integral part of young people’s everyday lives and offer an interactive digital environment that can be incorporated into sport training to support engagement and skill development. **Methods**: A total of 40 male basketball players aged 12–14 years participated in this quasi-experimental study. Participants were allocated by existing school teams, with one team assigned to the experimental group (*n* = 20) and the other to the control group (*n* = 20). Both groups completed a six-month training period consisting of three sessions per week. Hand–eye coordination and dribbling-related performance were evaluated using two standardized mobile-application-based field tests with both hands during initial and final assessments. The data were analyzed using mixed-design repeated-measures ANOVA, with time as the within-subject factor and group as the between-subject factor. **Results:** The mixed-design repeated-measures ANOVA showed significant time × group interactions for all assessed outcomes, indicating greater improvements in coordination performance and bilateral upper-limb performance in the experimental group compared with the control group. **Conclusions**: These results indicate that mobile-application-based training can be a practical and effective approach for developing coordination and supporting bilateral upper-limb performance in youth basketball players.

## 1. Introduction

Basketball is commonly described as a high-intensity intermittent sport that involves repeated explosive actions, including sprinting, jumping, sudden changes of direction, and frequent physical contact [1]. These movements take place in environments that change rapidly, requiring players to coordinate movement while simultaneously processing visual and tactical information [2]. Performance, therefore, relies not only on aerobic and anaerobic capacity, but also on the athlete’s ability to control movement and make decisions under time constraints.

In the context of human movement science, coordination is generally described as the organization and regulation of movement patterns, a concept discussed extensively within biomechanics and motor control research [3]. In basketball, this concept is particularly relevant because game situations are influenced by multiple factors, such as ball position, player movement, and remaining game time. Moreover, because play shifts rapidly between offense and defense, athletes must constantly adapt their movement patterns with precise timing [4,5]. For that reason, coordination is a key determinant of technical performance. Well-developed coordination supports more efficient skill execution, optimized movement mechanics, and more rapid adjustment to dynamic conditions [6,7,8]. This not only enhances technical accuracy but also supports better management of physical load [9].

Between the ages of 12 and 14, players undergo a notably favorable period for improving coordination, as the nervous system is highly responsive to motor learning [10,11]. Coordination development, however, is not limited exclusively to adolescence, as structured coordination training has also been shown to produce beneficial effects in younger school-aged children, including improvements in motor and cognitive functions [12]. However, the 12–14-year age range also corresponds to early adolescence, when rapid growth and maturational changes may temporarily affect movement fluency and produce a degree of motor awkwardness, especially in boys [13]. In this context, structured coordination training may help players adapt to these developmental changes while refining visual–motor control and basketball-specific movement patterns. Targeted coordination training may accelerate skill development, offering the players a good technical base to be further developed [14]. For this reason, coordination training should represent a key element of youth basketball preparation during early adolescence. In basketball, coordination is closely linked to bilateral upper-limb use, because repeated execution of ball-handling, passing, and shooting tasks with a preferred hand may reinforce unequal motor patterns and contribute to functional differences between the two upper limbs.

Limb dominance (functional preference for one side during skilled tasks) is commonly attributed to neural lateralization [15,16]. In basketball, despite the need for coordinated use of both arms [17], players frequently rely more on their dominant hand when executing key technical skills such as ball handling, passing, and shooting [18,19,20]. However, as the competitive level increases, athletes tend to demonstrate greater involvement of the non-dominant limb [18], suggesting that balanced bilateral ability contributes to higher performance standards. Inter-limb asymmetries in strength and power have been associated with modified movement mechanics and may contribute to injury risk [21,22]. For this reason, identifying and reducing upper-limb asymmetries should be considered an important objective in structured basketball training, especially in youth development stages.

Modern technologies are increasingly used in basketball to support training monitoring and performance analysis [23,24]. Advanced systems such as motion capture devices, wearable trackers, and specialized analysis software gives substantial information about movement quality, workload, and physical responses. Although they offer several advantages, these tools often involve substantial financial investment and technical support, which limits their availability for many smaller clubs, school programs, or families in lower resource environments. By comparison, smartphones are widely available and include cameras and inertial sensors that can be used for field-based assessment. With the help of freely available applications, coaches and players can review technique, measure time, and monitor certain physical indicators without additional equipment. Although they cannot replace laboratory systems, they may provide actionable feedback in routine practice, especially in youth development.

Nevertheless, mobile applications also have limitations. Their measurements may be less precise than those obtained using laboratory-based systems, motion capture, or specialized sport technologies, and performance can be influenced by device characteristics such as screen size, touch sensitivity, refresh rate, and application design. In addition, app-based coordination tasks may not fully reproduce the perceptual and tactical complexity of real basketball situations. Therefore, mobile applications should be considered practical complementary tools rather than replacements for validated laboratory or sport-specific assessment systems.

An essential advantage of mobile-based training tools is the possibility of delivering instantaneous feedback [25]. Instant visual or numerical information allows athletes to correct technical errors in real time, which is known to develop motor learning efficiency [26]. Many apps incorporate gamified features (scores, levels, progress metrics), which may enhance adherence. Such features can increase engagement and sustain motivation, particularly in youth athletes who respond positively to interactive and goal-oriented tasks.

Digital tools also contribute to greater training standardization [27]. Recent research confirms that app-based feedback and augmented visual information can positively influence skill development, especially when combined with repetitive practice [28,29,30]. Despite these aspects, there remains a limited number of studies examining effects of mobile application training programs with coordination development, and limb symmetry in youth basketball players.

Most studies in youth basketball training have focused on conventional approaches such as resistance training, plyometric exercises, and general coordination programs [31,32,33,34,35]. Although these methods have demonstrated benefits for physical and technical performance, less attention has been given to accessible digital tools that can be integrated directly into routine training environments. In particular, limited evidence is available regarding mobile-application-based coordination training in young basketball players and its effects on right- and left-hand coordination performance during basketball-specific tasks.

Therefore, the present study aimed to examine whether adding a structured mobile-application-based coordination component to regular basketball training would improve coordination performance in 12–14-year-old male basketball players. Specifically, the study evaluated changes in right- and left-hand performance using two standardized mobile-application-based field tests before and after a six-month intervention. Based on previous evidence showing that technology-assisted feedback, visual stimuli, and structured coordination training can support motor learning and sport-specific skill development [25,26,27,28,29,35,36], we hypothesized that the experimental group would demonstrate greater improvements than the control group in both Tapd coordination and hand–eye coordination test performance. These improvements were expected primarily in standardized coordination tests and training drills, rather than being directly evaluated in actual competitive match performance.

## 2. Materials and Methods

### 2.1. Participants

This study focused on young, active male basketball players competing at the national junior level in Romania. All participants were registered athletes taking part in organized club training and competitions. The group included players aged between 12 and 14 years, corresponding to the U13 and U14 categories, and was divided into two groups: an experimental group (*n* = 20) and a control group (*n* = 20), each including both U13 and U14 players. Participants were not randomly assigned to groups. The experimental and control groups were formed from two existing basketball teams from the same school, both training under the supervision of the same coach. One team was assigned as the experimental group and the other as the control group. Therefore, the study followed a quasi-experimental design rather than a randomized controlled design. The experimental group had a mean age of 13.25 ± 0.72 years, while the control group had a mean age of 12.95 ± 0.69 years. No statistically significant difference was observed between groups for age, t(38) = 1.352, *p* = 0.184. Although the groups were comparable in chronological age and anthropometric characteristics, biological maturity indicators such as Tanner stage or maturity offset were not assessed. Therefore, chronological age was used only to describe the sample, and no direct conclusion can be made regarding biological maturation status. The experimental group presented average anthropometric values of 158.65 ± 9.94 cm in height, 47.50 ± 10.72 kg in body mass, and 157.25 ± 11.54 cm in wingspan. The control group showed mean values of 156.75 ± 6.03 cm for height, 46.20 ± 8.25 kg for body mass, and 154.95 ± 6.89 cm for wingspan. Independent samples *t*-tests showed no statistically significant differences between the experimental and control groups for height (*p* = 0.469), body mass (*p* = 0.670), or wingspan (*p* = 0.450), indicating that the groups were comparable in terms of anthropometric characteristics at baseline. All players were clinically healthy, actively involved in regular basketball training, and had previous competitive experience appropriate to their age category. The exclusion criteria were as follows: presence of acute injury or medical condition limiting participation in basketball training or testing; absence from either the initial or final testing session; irregular participation in the training program; lack of parental/legal guardian consent; and withdrawal from the study at any point. Players who did not meet the inclusion criteria were not included in the final analysis. Throughout the study period, both experimental and control groups followed structured training programs adapted to their developmental level, with the experimental group additionally participating in mobile-application-based coordination training. All procedures were conducted in accordance with the ethical principles outlined in the Declaration of Helsinki. A sensitivity power analysis was performed using G*Power 3.1.9.7 to assess the robustness of the available sample size. For an independent samples *t*-test, with two groups of 20 participants, a two-tailed alpha level of 0.05, statistical power of 0.80, and an allocation ratio of 1:1, the available sample size was sufficient to detect large between-group effects of Cohen’s d = 0.901. Therefore, the final sample of 40 players was considered acceptable for detecting large effects in this exploratory intervention study.

### 2.2. Study Design

This study used a quasi-experimental pre-test/post-test design with two parallel groups: an experimental group and a control group. Both groups were evaluated before and after the six-month training period using the same testing procedures. The experimental group completed additional mobile-application-based coordination drills, whereas the control group followed the regular basketball training program without the use of mobile applications.

### 2.3. Testing Procedure

Before the official testing, all participants received standardized instructions and a demonstration of each task. They also completed a brief familiarization trial for each test to ensure understanding of the procedure and to reduce potential learning effects during the recorded assessment.

**Figure 1:**
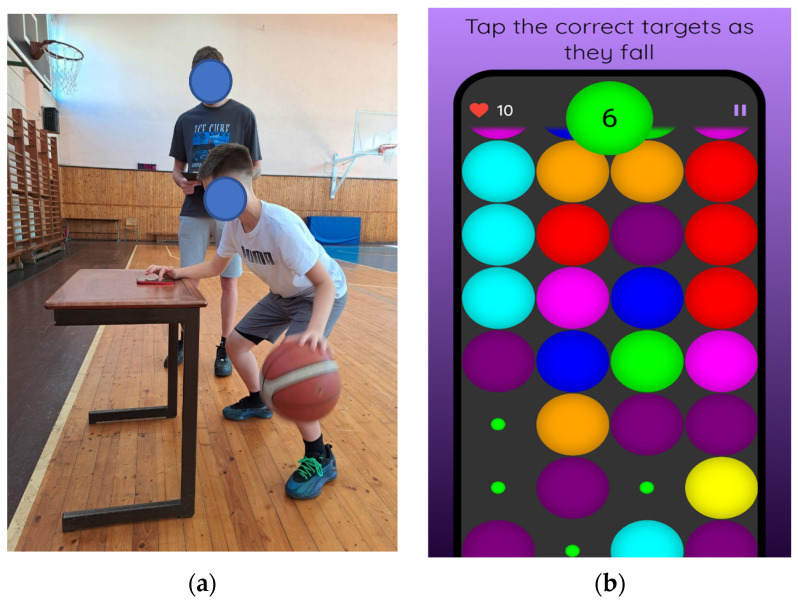
Tapd coordination test. (**a**)—player and coach position; (**b**)—application interface.

Test 1. Tapd coordination test (Figure 1).

This test used “Tapd: A Coordination Game,” which is a mobile application available on any device using iOS or Android and it was designed to assess hand–eye coordination under dynamic conditions. From a stationary dribbling position while maintaining the fundamental basketball stance, the student was required to tap the circle corresponding to the target color displayed at the top of the screen. Simultaneously, multiple circles of different colors descended on the screen at a speed that increased progressively over time. The target color changed each time the student achieved a score of 10 points (i.e., 10 correct selections). One point was awarded for each correct tap, while the selection of an incorrect color immediately terminated the test. Therefore, the test had no fixed duration, as its length depended on each participant’s ability to maintain correct responses before the first error. The final score was represented by the total number of correctly selected targets achieved during the test. The smartphone was placed in front of the student on a table at 80 cm height. To reduce the potential influence of fatigue, all participants were first tested with one hand, and only after all players had completed that trial was the test repeated with the opposite hand. This procedure provided a recovery interval between dominant- and non-dominant-hand testing.

**Figure 2:**
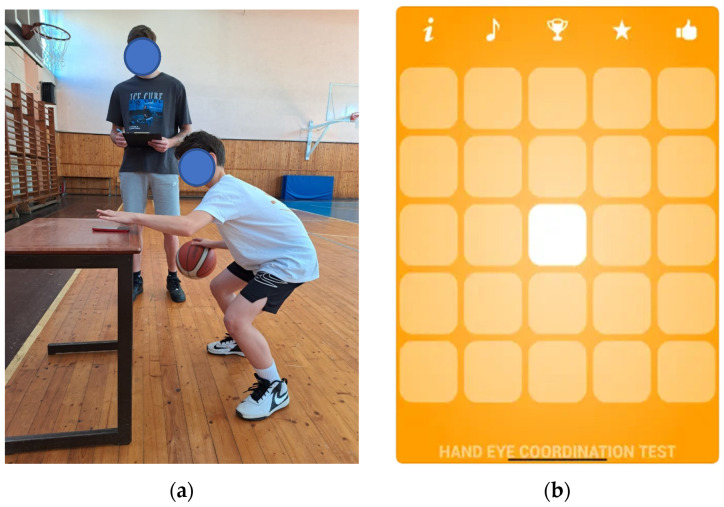
Hand–eye coordination test. (**a**)—player and coach position; (**b**)—application interface.

Test 2. Hand–Eye coordination test (Figure 2).

This test used the “Hand eye test” mobile application available on any iOS or Android device, and unlike the previous assessment, it was performed under a fixed time constraint, 30 s. Participants were in fundamental basketball stance, with a table with height of 80 cm in front of them and a smartphone placed on it. During the test, participants performed a stationary basketball dribble using one hand, while the opposite hand was used to interact with the touchscreen. The application interface displayed a grid of squares, with only one square illuminated in white at any given time. Participants were instructed to tap the illuminated square as quickly and accurately as possible. Following each correct tap, the illuminated square changed its position randomly on the screen, requiring continuous visual scanning and rapid hand–eye coordination. The total number of correctly tapped illuminated squares registered within 30 s represented the participant’s final score. Upon completion of the test the dribbling hand was changed.

No separate penalty system was applied for temporary loss of ball control during testing. If a participant lost control of the ball, the test continued, and the player was instructed to regain control as quickly as possible while maintaining the task. Such errors were reflected directly in performance, as they reduced the number of correct responses achieved during the test. The two mobile-application-based tests offer a practical way to evaluate key basketball skills in young players. They not only measure dribbling control but also provide insight into hand–eye coordination, reaction speed, and the ability to coordinate multiple movements at once. By testing both hands separately, the protocol allowed bilateral upper-limb performance to be described, although it did not provide a formal asymmetry index. Overall, these tests serve as both an assessment tool and a basis for designing training programs aimed at improving coordination, reaction ability, and functional symmetry in 12- to 14-year-old basketball players.

The mobile applications were not used as independent diagnostic instruments, but as components of a standardized, basketball-specific field-testing protocol designed to evaluate coordination performance under practical training conditions. To ensure procedural consistency, the same device placement, task instructions, scoring rules, testing environment, and evaluator supervision were applied for all participants during both initial and final testing. To the authors’ knowledge, no published validity or reliability coefficients are currently available for these applications under the exact testing conditions used in the present study. Therefore, the results should be interpreted as performance outcomes from standardized field-based coordination tasks rather than as measurements derived from formally validated laboratory instruments. This aspect is acknowledged as a methodological limitation and was considered when interpreting the findings.

### 2.4. Research Design

The study was organized as follows: initial testing was carried out between Monday, 9 September 2024, and Friday, 13 September 2024. Following this, a mobile-application-based training program was implemented over a six-month period, aimed at improving hand–eye coordination, dribble control, and bilateral upper-limb performance. The final testing took place from Monday, 10 March 2025, to Friday, 14 March 2025.

The experimental training program was designed for 12- to 14-year-old basketball players and involved exercises using mobile applications to support the development of coordination and bilateral upper-limb symmetry. Players in both experimental and control groups trained three times per week for 90 min starting each session with 20 min warm-up. Following the warm-up, the control group engaged in conventional basketball drills, whereas the experimental group completed the exercises included in the mobile-application-based training program.

### 2.5. Training Procedure

The training program implemented in this study was designed to develop both basketball-specific skills and the coordinative abilities, very important for young players. Over the six-month training program, participants took part in three weekly sessions, each lasting 90 min and beginning with a 20 min warm-up. During each session, players performed a selection of five drills from a total of 40 exercises, ensuring both variety and consistent practice. This approach allowed repeated exposure to key skills while progressively reinforcing technical and coordinative abilities. To improve replicability, the intervention was structured according to the FITT-VP model. Frequency: the program was implemented three times per week over a six-month period. Intensity: the coordinative intensity was controlled through task complexity, including visual and auditory stimuli, reaction speed demands, bilateral hand use, and progressive increases in difficulty within the mobile applications. Time: each training session lasted 90 min, including a 20 min warm-up, with the mobile-application-based coordination drills performed after the warm-up. Type: the intervention consisted of basketball-specific coordination drills combined with mobile applications targeting hand–eye coordination, reaction ability, dribbling control, movement combination, and upper-limb symmetry. Volume: during each session, players completed five drills selected from a pool of 40 exercises. Progression: progression was achieved by increasing task difficulty, varying stimuli, alternating dominant and non-dominant hand execution, and combining dribbling with visual or auditory response tasks.

The control group trained with the same weekly frequency and session duration as the experimental group, consisting of three 90 min basketball sessions per week over the six-month period. Each session began with a 20 min warm-up, followed by conventional basketball drills focused on technical and tactical development, including dribbling, passing, shooting, footwork, defensive movements, and small-sided game situations. The control group did not perform mobile-application-based drills and did not receive app-based visual or auditory feedback during training. This approach ensured that both groups had comparable training volume, while the main difference between groups was the inclusion of the mobile-application-based coordination component in the experimental group.

The drills were designed to address multiple objectives simultaneously, combining the development of basketball-specific skills with the enhancement of coordinative abilities. Each exercise was structured to engage several key components, including:

- Hand–eye coordination (28 exercises): improving the synchronization between visual cues and hand movements, important for dribbling, passing, and shooting.
- Reaction ability (40 exercises): aimed at reducing response time to visual or auditory signals, a key factor during fast gameplay.
- Dribbling control (24 exercises): enhancing control, precision, and adaptability when handling the ball under dynamic conditions.
- Ability to combine movements (18 exercises): developing fluid execution of multiple actions, integrating dribbling, passing, and changes of direction.
- Upper-limb symmetry and asymmetry (32 exercises): assessing and improving bilateral coordination to ensure balanced development of both arms.

The program incorporated four mobile applications—Tap Of War, Color Reaction, Sound Buttons/Instant Buttons, and Lights: A Memory Game—which provided interactive and cognitively engaging exercises. These applications offered real-time feedback, allowed objective measurement of reaction speed, hand–eye coordination, and bilateral limb performance, and increased the motivation of the players. By combining mobile app–based drills with traditional basketball exercises, the program created a dynamic training environment that developed both technical skills and coordination in players aged 12–14 years old.

### 2.6. Statistical Analysis

A sensitivity power analysis was performed using G*Power 3.1.9.7 to assess the robustness of the available sample size. For an independent samples *t*-test, with two groups of 20 participants, a two-tailed alpha level of 0.05, statistical power of 0.80, and an allocation ratio of 1:1, the available sample size was sufficient to detect large between-group effects of Cohen’s d = 0.901.

Data were analyzed using IBM SPSS Statistics version 26. Descriptive statistics were calculated for all variables, including minimum, maximum, mean, standard deviation, variance, kurtosis, and coefficient of variation. Baseline differences between the experimental and control groups for age and anthropometric variables were examined using independent samples Student’s *t*-tests. Intervention effects on coordination performance were analyzed using mixed-design repeated-measures ANOVA, with time as the within-subject factor, consisting of initial and final testing, and group as the between-subject factor, consisting of the experimental and control groups. The time × group interaction was used to determine whether changes from initial to final testing differed between groups. Statistical significance was set at *p* < 0.05. Effect sizes for the ANOVA were reported using partial eta squared (η^2^p).

Right- and left-hand performances were analyzed as separate outcome variables for each coordination test. A separate asymmetry index was not calculated in the present study; therefore, the statistical analysis does not provide a direct numerical quantification of inter-limb asymmetry. Accordingly, the interpretation of the findings was restricted to changes in bilateral upper-limb coordination performance, based on the separate analysis of right- and left-hand outcomes across time and groups.

## 3. Results

### 3.1. Descriptive Statistics

Descriptive statistics for the Tapd coordination test are presented in Table 1. Overall, both groups showed increases from initial testing to final testing; however, the magnitude of improvement appeared greater in the experimental group. In the experimental group, mean scores increased for both the right and left hands, while the control group showed only limited changes across the same period. The coefficients of variation remained relatively low in most conditions, suggesting acceptable within-group consistency. These descriptive findings indicate a stronger improvement trend in the experimental group, which was further examined using repeated-measures ANOVA.

Descriptive statistics for the hand–eye coordination test are presented in Table 2. Similar to the Tapd coordination test, the experimental group demonstrated larger mean increases from initial to final testing than the control group for both hands. The left-hand results showed particularly clear improvement in the experimental group, while the control group presented smaller changes. Variability was higher in the right-hand performance of the experimental group at final testing, indicating greater individual differences in response to the intervention. These descriptive results suggest favorable changes in the experimental group, which were subsequently tested through repeated-measures ANOVA.

When considered together, the descriptive results show a consistent pattern of greater improvement in the experimental group across both coordination tests. However, because descriptive statistics alone do not determine whether the changes differed significantly between groups, the main intervention effects were analyzed using mixed-design repeated-measures ANOVA.

### 3.2. Mixed-Design Repeated-Measures ANOVA

The repeated-measures ANOVA from Table 3, showed significant main effects of time for all four outcomes, indicating that performance improved from initial to final testing across the sample. More importantly, significant time × group interactions were observed for each variable, showing that the magnitude of improvement differed between the experimental and control groups. In all outcomes, greater gains were observed in the experimental group after the six-month mobile-application-based training program.

For the Tapd coordination test, significant time × group interactions were observed for both the right and left hands, with large partial eta squared values. This suggests that the intervention had a strong effect on coordination performance under progressively increasing task difficulty. Similar results were found for the hand–eye coordination test, where significant time × group interactions were also identified for both hands, indicating greater improvement in the experimental group in time-constrained coordination tasks.

Overall, the ANOVA results support the effectiveness of the mobile-application-based training program in improving coordination performance. The significant interaction effects suggest that the observed improvements cannot be explained only by repeated testing or regular basketball practice, as greater improvements were found in the experimental group.

## 4. Discussion

The present study examined whether a six-month mobile-application-based training program could enhance coordination performance in 12–14-year-old basketball players. Following the revised statistical analysis, the mixed-design repeated-measures ANOVA showed significant time × group interactions for all assessed outcomes, indicating that the experimental group improved significantly more than the control group from initial to final testing. These findings support the effectiveness of the mobile-application-based intervention in improving coordination performance in both the Tapd coordination test and the hand–eye coordination test.

The findings of this study align with research confirming that technology-assisted training can improve basketball-related coordination [4,36], especially when immediate feedback is provided by the executed drills [37]. Also, several studies that have used specialized light-stimulus systems [26,36,38] support the direction of present results. These findings are consistent with the improvements observed in our experimental group across both assessments, particularly because our intervention similarly focused on fast responses to visual stimuli in basketball specific drills. Compared with FitLight or other AR systems, the present intervention was based on widely accessible smartphones and low-cost applications. This approach requires minimal equipment and financial investment, making it easier to implement in school or club settings with limited resources. The smartphone applications used in the present study should not be interpreted as equivalent to validated laboratory-based or specialized sport assessment tools. Instead, they were implemented as practical field-based tools within a standardized basketball-specific protocol. Although digital tapping and hand–eye coordination tools have shown promising validity and reliability in other contexts, no direct validation studies were identified that compare the exact applications used in the present study with established coordination assessment instruments under the same basketball-specific conditions. Therefore, the findings should be interpreted as changes in standardized field-based coordination task performance rather than as measurements equivalent to validated laboratory assessments.

While direct basketball app–based intervention studies targeting upper-limb symmetry specifically appear relatively limited in the basketball literature, related smartphone-based training work in youth sport demonstrates that short, structured smartphone game interventions can produce measurable changes in cognitive performance [39]. Overall, previous technology-supported interventions suggest that shared elements such as immediate feedback [40], progressively increasing task demands [4], and repeated practice under perceptually demanding conditions [41] may explain their effectiveness. These components likely contributed to the coordination improvements and the reduction in performance differences between the dominant and non-dominant hands observed in the experimental group. Beyond technology assisted interventions, several studies have reported improvements in coordination and technical performance following structured coordination-based training programs in youth basketball players [42,43]. Programs incorporating reaction drills, perceptual tasks, or combined cognitive motor exercises have been shown to enhance dribbling efficiency, agility, and decision-making [44]. These findings support the view that coordination development during early adolescence responds positively to structured and repetitive stimuli. However, most of these investigations relied on traditional drills or specialized equipment rather than widely available mobile applications. The present study extends this line of research by demonstrating that similar improvements can be achieved using accessible smartphone-based tools.

Research examining inter-limb asymmetries in basketball has predominantly focused on lower-limb strength and power differences, often linking asymmetries to performance limitations or injury risk [45,46]. In contrast, upper-limb bilateral performance, particularly in relation to ball handling and coordination, has received less attention. In the present study, right- and left-hand performances were analyzed separately, and both showed significant time × group interactions, indicating greater improvements in the experimental group. However, a formal asymmetry index was not calculated, and no separate statistical analysis of inter-limb asymmetry was performed. Therefore, these findings should be interpreted as improvements in bilateral upper-limb coordination performance rather than as direct evidence of reduced inter-limb asymmetry.

The findings of this study have practical relevance for youth basketball training, particularly in school and club settings where access to advanced technological systems may be limited. Coaches may integrate mobile-application-based coordination drills as a complementary component of regular practice to stimulate hand–eye coordination, reaction ability, dribbling control, and bilateral upper-limb performance. These drills can be implemented after the warm-up or within technical training segments, using short tasks that combine ball handling with visual or auditory stimuli.

From a coaching perspective, mobile applications may offer a low-cost and accessible way to increase task variety, provide immediate feedback, and maintain player motivation through interactive and game-like exercises. However, such tools should not replace traditional basketball instruction, coach observation, or validated assessment systems. Instead, they should be used as supplementary training resources within a structured and progressive training plan, especially for young players who are developing fundamental coordination and ball-handling skills.

Taken together, the current findings contribute to the growing literature supporting coordination-focused training in youth basketball. Importantly, this study demonstrates that meaningful improvements in both coordination performance and upper-limb symmetry can be achieved through cost-effective and scalable methods. This may be particularly relevant for school programs and clubs operating without access to advanced technological systems.

### Limitations

A methodological limitation of the testing protocol is that the smartphone was placed horizontally on a table, which may have encouraged a downward visual focus. In actual basketball situations, players are required to maintain visual awareness of teammates, opponents, and spatial cues while controlling the ball. Therefore, future studies should consider mounting the mobile device on a vertical surface or at eye level to better reproduce basketball-specific attentional demands during coordination testing. A further limitation is that biological maturity was not assessed using Tanner stage, maturity offset, or other maturation indicators. This is relevant because players of the same chronological age may differ in biological maturation, which may influence coordination, motor control, and training responsiveness. Future studies should include maturity-related measures to better control for developmental differences in youth basketball players.

## 5. Conclusions

The present study showed that a six-month mobile-application-based training program was associated with greater improvements in coordination performance in 12–14-year-old basketball players compared with regular basketball training alone. The mixed-design repeated-measures ANOVA demonstrated significant time × group interactions for both the Tapd coordination test and the hand–eye coordination test, indicating larger improvements in the experimental group from initial to final testing.

These findings support the use of mobile applications as a practical and low-cost complement to traditional basketball training for developing hand–eye coordination and bilateral upper-limb performance in youth players. However, because match performance was not assessed and no specific asymmetry index was calculated, the results should be interpreted in relation to standardized coordination tasks rather than direct competitive performance or definitive changes in inter-limb asymmetry. Future studies should include larger samples, both sexes, match-related performance indicators, and direct measures of upper-limb asymmetry.

## Figures and Tables

**Table 1:** Descriptive statistics Tapd coordination test.

Test	Group	Phase	N	Min.	Max.	Mean	Std. Dev.	Variance	Kurtosis		CV
									Statistic	Std. Error	
**Tapd coordination test, right**	EG	It	20	32.00	42.00	35.650	3.066	9.397	−0.602	0.992	8.60%
**Tapd coordination test, right**	EG	Ft	20	42.00	57.00	48.350	3.870	14.976	0.015	0.992	8.00%
**Tapd coordination test, left**	EG	It	20	40.00	55.00	46.350	3.870	14.976	0.015	0.992	8.35%
**Tapd coordination test, left**	EG	Ft	20	53.00	72.00	59.650	4.568	20.871	1.760	0.992	7.66%
**Tapd coordination test, right**	CG	It	20	35.00	41.00	37.600	2.326	5.411	−1.877	0.992	6.19%
**Tapd coordination test, right**	CG	Ft	20	36.00	42.00	39.350	2.346	5.503	−1.533	0.992	5.96%
**Tapd coordination test, left**	CG	It	20	44.00	58.00	48.800	4.200	17.642	−0.017	0.992	8.61%
**Tapd coordination test, left**	CG	Ft	20	42.00	63.00	49.900	5.139	26.411	1.069	0.992	10.30%

Note: EG = experimental group; CG = control group; It = initial testing; Ft = final testing; N = number of participants; Min. = minimum value; Max. = maximum value; Std. Dev. = standard deviation; CV = coefficient of variation.

**Table 2:** Descriptive statistics hand–eye coordination test.

Test	Group	Phase	N	Min.	Max.	Mean	Std. Dev.	Variance	Kurtosis		CV
									Statistic	Std. Error	
**Hand–eye test, right**	EG	It	20	4.00	11.00	8.150	2.254	5.082	−1.148	0.992	27.66%
**Hand–eye test, right**	EG	Ft	20	8.00	24.00	13.750	4.506	20.303	−0.236	0.992	32.77%
**Hand–eye test, left**	EG	It	20	9.00	15.00	10.600	1.667	2.779	1.058	0.992	15.73%
**Hand–eye test, left**	EG	Ft	20	14.00	23.00	18.100	2.100	4.411	0.264	0.992	11.60%
**Hand–eye test, right**	CG	It	20	6.00	13.00	10.050	1.701	2.892	0.320	0.992	16.92%
**Hand–eye test, right**	CG	Ft	20	7.00	14.00	10.950	1.986	3.945	−0.385	0.992	18.14%
**Hand–eye test, left**	CG	It	20	10.00	15.00	12.400	1.729	2.989	−1.444	0.992	13.94%
**Hand–eye test, left**	CG	Ft	20	11.00	17.00	13.700	1.625	2.642	−0.654	0.992	11.86%

Note: EG = experimental group; CG = control group; It = initial testing; Ft = final testing; N = number of participants; Min. = minimum value; Max. = maximum value; Std. Dev. = standard deviation; CV = coefficient of variation.

**Table 3:** Mixed-design repeated-measures ANOVA results for coordination performance.

Variable	Effect	F	df	*p*	η^2^p
**Tapd, right**	Time	218.502	1.38	<0.001	0.852
**Tapd, right**	Time × group	91.902	1.38	<0.001	0.707
**Tapd, left**	Time	115.513	1.38	<0.001	0.752
**Tapd, left**	Time × group	101.585	1.38	<0.001	0.728
**Hand–eye, right**	Time	66.396	1.38	<0.001	0.636
**Hand–eye, right**	Time × group	26.775	1.38	<0.001	0.413
**Hand–eye, left**	Time	133.134	1.38	<0.001	0.778
**Hand–eye, left**	Time × group	58.374	1.38	<0.001	0.606

Note: df = degrees of freedom; *p* = statistical significance level; η^2^p = partial eta squared; Time = comparison between initial and final testing; Time × group = interaction between testing moment and group.

## Data Availability

The data presented in this study are available on request from the first author due to ethical and data protection considerations.
